# High-Salt Diets, Intestinal Barrier, and Hypertension: A Mechanistic Review and the Promise of Dietary Therapy

**DOI:** 10.3390/nu17233688

**Published:** 2025-11-25

**Authors:** Wenhao Si, Yan Zhao, Yuhang Wu, Jiani Jiang, Hui Zheng, Yong Yang, Tao Zheng

**Affiliations:** School of Pharmacy, Hunan University of Chinese Medicine, Changsha 410208, China; kyoumachris613@gmail.com (W.S.); 17507380875@163.com (Y.Z.); 20233795@stu.hnucm.edu.cn (Y.W.); 20233794@stu.hnucm.edu.cn (J.J.); huizheng0104@163.com (H.Z.)

**Keywords:** high-salt diets, hypertension, gut microbiota, intestinal barrier, dietary therapy

## Abstract

Hypertension is a major public health problem worldwide, and high-salt diets are one of the main causes of hypertension. The intestinal mucosal immune system is the largest immune organ in vertebrates. Hypertension was associated with increased intestinal permeability and an inflammatory state. The bacterial communities attached to the intestinal mucosa played a significant role in the development and maturation of the autoimmune system, as well as inflammation and immunity to disease. In this review, we focused on the relationship between the impaired intestinal barrier and the development and progression of hypertension under the high-salt dietary pattern. We systematically reviewed how a high-salt diet caused hypertension by disrupting the intestinal mechanical, chemical, and microbial barriers, interacting with immunogenic isolevuglandin (IsoLG)-protein adducts and microbiota, and activating the mitogen-activated protein kinase (MAPK)/nuclear factor-kappa B (NF-κB) signaling pathway. Meanwhile, this review also summarizes the dietary therapy for hypertension, which involves supplementing natural antihypertensive substances and adjusting dietary patterns to repair the intestinal barrier and assist in lowering blood pressure. Such measures included supplementing plant-based foods, polyunsaturated fatty acids (PFAs), probiotics, prebiotics, food–medicine homologous substances (FMHS), vitamins, and minerals, as well as transforming high-salt dietary patterns into the dietary approaches to stop hypertension (DASH), the Mediterranean diet (MD), and the ketogenic diet (KD), with the aim of providing a reference for the occurrence, development, and dietary prevention and control of high-salt hypertension.

## 1. Introduction

Diet is a modifiable risk factor to which the human body is exposed throughout its lifetime. The risks that diet brings to human health are an important issue that exists globally [1,2]. Due to changes in dietary habits and lifestyles, processed foods and takeout foods have increasingly entered people’s lives, leading to excessive salt intake in many countries around the world, far exceeding the recommended intake levels. The first “Global Sodium Reduction Report” released by the World Health Organization in 2023 showed that the global average sodium intake is 4310 mg/day (10.78 g of salt per day), far exceeding the recommended limit of 5 g/d [3]. For example, in China, surveys have shown that the average daily salt intake of residents in some areas was 9.14–11.22 g/d, which was much higher than the recommended intake in dietary guidelines [4,5]. High-salt diets have become a common dietary pattern in most areas of China and are considered one of the significant public health issues worldwide that induce sub-healthy conditions.

The “International Classification of Diseases” defines hypertension as a systemic chronic inflammatory disease. There are over one billion people worldwide suffering from hypertension. The prevalence of hypertension doubled globally between 1990 and 2019, affecting ~25% of men and ~20% of women [6,7]. According to six rounds of surveys conducted in China in 2018, there were 274 million adults aged 18–69 with hypertension, and the standardized prevalence rate was 24.7% [8]. Hypertensive patients were more prone to suffer from cardiovascular and cerebrovascular, and renal diseases, so controlling blood pressure was important to reduce the morbidity and mortality of hypertension-related complications [9,10,11]. High-salt diets have been positively linked to hypertension, and research from the early 1900s revealed that diets low in salt could help treat hypertension [12]. An epidemiological study involving 18 countries and more than 100,000 people showed a significant correlation between daily salt intake and blood pressure [13]. For every additional 1 g of sodium intake, SBP and DBP increased by 2.11 mmHg and 0.78 mmHg, respectively [14]. Compared with the group with sodium intake more than 2 g/d, the low-salt diet group with sodium intake less than 2 g/d had a decrease in SBP and DBP by 3.47 mmHg and 1.88 mmHg [15]. Although the link between a high-salt diet and hypertension has been confirmed by both clinical and experimental research, the underlying mechanisms remain unclear.

The intestinal mucosal immune system is the largest immune organ in vertebrates and can effectively defend the digestive tract against pathogens, endotoxins, and antigens. An intact intestinal barrier, which is made up of four main parts—mechanical, biological, immunological, and chemical—is essential for intestinal health and homeostasis. The gut microbiota, including luminal microbiota and intestinal mucosal microbiota, is the main component of the intestinal barrier and crucial for the maintenance of the intestinal barrier function. Impaired intestinal barrier function is a major pathophysiology of chronic inflammatory disorders and can cause systemic or local immune responses. The development and incidence of hypertension are significantly influenced by inflammation and the immune system, and the pathophysiological mechanism of hypertension is linked to the function of the intestinal barrier. Intestinal mucosal microbiota has been shown to have an impact on immune system development, preserve the intestinal barrier’s integrity, and prevent infections [16,17]. If the gut microbiota is imbalanced, it could disorganize the intestinal system, leading to an increase in intestinal permeability and ultimately disrupting the intestinal barrier function [18,19,20]. The gut microbiota can change as a result of both endogenous and external influences, and the organism’s food and drugs impact it. Under a long-term high-salt dietary pattern, the intestinal barrier function was impaired, leading to bacterial translocation, allowing bacterial products in the intestine to enter the circulatory system and activate systemic inflammation. A chronic low-grade inflammatory process is necessary for the development of hypertension. Chronic low-grade inflammatory processes are required for the development of hypertension, and the process of intestinal barrier impairment causes endothelial cell dysfunction and vascular sclerosis, activating pathologic vascular inflammation, which in turn causes bacteria and their metabolites to enter the bloodstream circulation. This worsens the pathologic process of hypertension, which is linked to the pathogenesis of hypertension. In a chronic high-salt dietary pattern, impaired intestinal barrier function results in bacterial translocation, which permits bacterial products in the gut to enter the circulatory system and trigger systemic inflammation [21,22]. Hypertension, in turn, can also affect the composition and function of the gut microbiota, altering the immune function and barrier integrity of the gut [16,17]. Therefore, the normalization of intestinal barrier function is closely related to the pathogenesis of high salt diet-induced hypertension.

“It can nourish health and cause disease” is a concise way to describe the dialectical link between diet and health, which is the most fundamental and significant physiological necessity of an individual. Excessive salt intake is closely linked to the onset of hypertension, and while regular medication is necessary to temporarily lower blood pressure, the side effects and expense of medications will increase the burden on patients. Instead, non-pharmacological treatments and the combined use of multiple approaches could effectively control blood pressure levels in hypertensive patients. Dietary therapy is a crucial component of non-pharmacologic treatment, and it can effectively lower blood pressure, particularly in people with early-stage moderate hypertension [23]. Therefore, dietary therapy has been shown to be a safe treatment option for managing hypertension, making it a significant tool for managing chronic diseases. It has been recognized as a sustainable hypertension intervention approach with evidence-based medical backing because of its efficacy in preventing adverse effects, including drug tolerance and physiological dependence, in clinical applications. The mechanisms by which a high-salt diet causes hypertension are methodically outlined in this review. These include the disruption of the intestinal mechanical, chemical, and microbial barriers as well as the stimulation of the immune system by excessive dietary sodium, which altered immunogenic IsoLG-protein adducts and gut microbiota and activated the MAPK/NF-κB pathway. Furthermore, this review also summarizes the dietary therapies for hypertension, such as supplementation with plant-based foods, PFAs, probiotics, and prebiotics; FMHS; or transformation of a high-salt diet pattern into dietary patterns like the DASH, MD, and KD. The mechanisms by which these hypertension dietary therapies lower blood pressure are also summarized in order to provide a reference for the occurrence, development, and dietary control of high-salt hypertension.

## 2. Materials and Methods

**Figure 1:**
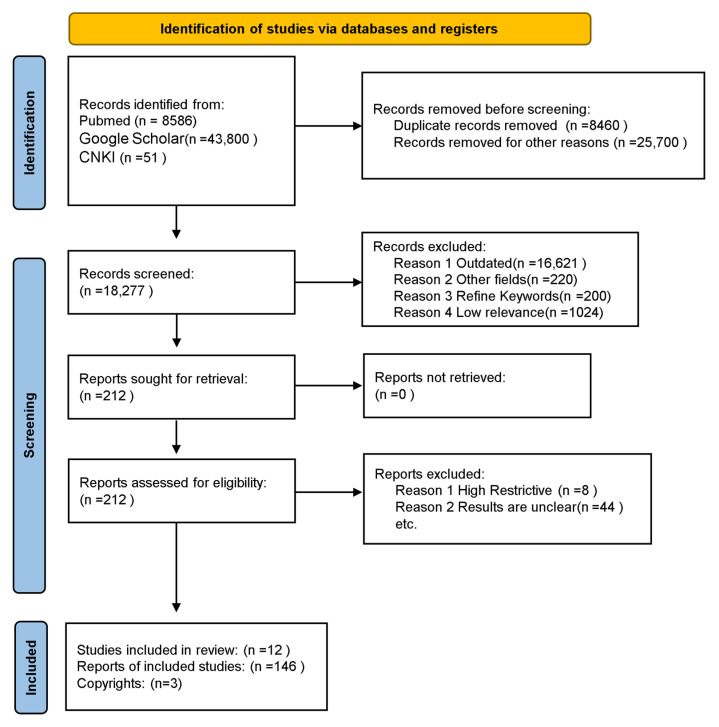
Flow PRISMA diagram of the screening and selection procedure.

Literature searches were conducted using PubMed, Google Scholar, and CNKI. Additionally, the reference lists of these articles were reviewed. Relevant literature was also retrieved by accessing institutional websites. EndNote 2020 was used for literature management. Given the interdisciplinary nature of the articles, search terms included combinations of “high-salt diet,” “intestinal barrier,” “hypertension,” and “dietary patterns.” Data extraction was performed by screening titles and selecting abstracts of relevant articles. Full texts were obtained if abstracts met the inclusion criteria (Figure 1).

## 3. High-Salt Diets and Hypertension

Salt is the most basic and commonly used food in people’s daily life, and it is used in daily cooking and seasoning, the food processing industry, etc. Salt provides sodium, and reasonable intake is important for maintaining plasma volume and acid–base balance, promoting protein and carbohydrate metabolism, and muscle contraction in the human body [24]. However, excessive salt intake will jeopardize human health [25,26]. With the rapid development of the food industry, the main source of salt has shifted from household cooking salt to processed foods. Salt, along with sugar and fat, is one of the three key ingredients in the food industry’s processing of foods. In order to satiate consumer pleasure and stimulate taste, food manufacturers would add huge amounts of all three elements to their products [27]. According to WHO 2023 documents, (1) the United States, as a representative country of the “high-salt food processing” model, has an average sodium intake of 3492 mg/day (8.9 g of salt per day); (2) China, as a country following the “traditional high-salt cooking” model, has an average sodium intake of 6954 mg/day (17.7 g of salt per day); (3) Poland, where the traditional diet includes substantial amounts of cured meats, sausages, and sauerkraut, has an average sodium intake of 4357 mg/day (11.1 g of salt per day); and (4) the United Kingdom implemented voluntary salt reduction initiatives from 2003 to 2011, achieving an average sodium intake of 2780 mg/day (7.1 g of salt per day) [3,28]. The Dietary Guidelines for Chinese Residents (2022) recommended a salt intake of 5 g/d for adults, but in many regions of China, the salt intake far exceeds this figure [4]. Table 1 presents a systematic summary and meta-analysis (2019) study that precisely estimates the average sodium intake of Chinese residents based on 24 h urine sodium content data. It is inferred from the sodium content in urine that the sodium intake of almost all age groups exceeds the standard [5]. Apart from seasonings, the primary sources of excessive sodium intake include snacks, fast meals, and pickled salted vegetables [29,30]. It is evident that people get their salt from a variety of foods, and regular exposure to takeout or prepackaged foods might result in high sodium intake. The change in dietary structure has led to excessive salt intake among residents in many areas. A high-salt diet has become a normalized dietary pattern in many regions [29,31,32,33].

Dietary pattern analysis is a potential method for determining the relationship between diet and chronic disease risk. Epidemiological studies on different dietary patterns and hypertension risk have shown that animal-based foods and high-salt diets were directly associated with an increased risk of hypertension [34]. However, salt intake is independently linked to the risk of hypertension [35]. For every additional gram of sodium intake, systolic blood pressure increases by 2.11 mmHg and diastolic blood pressure increases by 0.78 mmHg [14]. Hypertension is the most obvious and serious health concern connected with a high-salt diet. It is the most frequent chronic disease and one of the leading causes of serious cardiovascular and cerebrovascular diseases, which can result in disability or death. In populations where salt intake surpasses 24 g/day, hypertension prevalence might reach 42.5% [36]. The global prevalence of hypertension is increasing year after year, with younger age groups being most affected [37,38]. Hypertension’s pathogenesis is not fully understood due to its complex and diverse causes, which may include multiple complex physiological processes such as kidney function, vascular responses, hormonal regulation, neurological influences, genetic factors, changes in gut microbiota, and immune system-related regulation [39]. A high-salt diet increases the prevalence of this chronic condition [39]. Hypertension and its complications have a huge medical and financial impact on society, families, and individuals.

## 4. Intestinal Barrier and Gut Microbiota

The intestinal barrier function is mainly composed of intestinal epithelial cells, including absorptive intestinal cells, mucus-producing goblet cells, Paneth cells that produce antibacterial peptides, and enteroendocrine cells that produce hormones [40]. The intestinal mucosa is a layer of tissue that lines the inner wall of the intestine. It is exposed to gut microbiota and foreign pathogenic microorganisms and acts as the first line of defense for the intestinal immune system. The intestinal mucosa’s epithelial barrier consists of commensal bacteria, peptides, and antimicrobial proteins that prevent foreign colonization. The submucosa of the intestine contains dendritic cells (DCs), macrophages, innate lymphoid cells, T lymphocytes, plasma cells, and other intrinsic layer cells [40]. Intestinal epithelial cells and lamina propria cells are important mediators of innate immunity in intestinal barrier function. This plays an important role in the study of inflammation caused by the gut microbiota and its mediation of chronic diseases such as hypertension. The Toll-like receptor 4 (TLR4) receptor could identify metabolites like lipopolysaccharide (LPS) made by Gram-negative bacteria (like *Escherichia coli*), which could cause vascular inflammation, endothelial dysfunction, and oxidative stress. These factors also contribute to the development of hypertension [18].

The gut microbiota affects the intestinal barrier function by directly or indirectly affecting the intestinal mucosa, which is the primary site of excess salt absorption in the body [41]. A high salt diet caused microecological changes in the gut microbiota, leading to dysbiosis and affecting the production of metabolites. More than 90% of the gut microbiota in a healthy human body is made up of Gram-negative bacteria from the phylum Bacteroidetes (mainly *Bacteroides* or *Prevotella*) and Gram-positive bacteria from the phylum Firmicutes (mainly *Clostridium* and *Lactobacillus*) [42]. It has significant ramifications for studies on hypertension brought on by a high-salt diet. Increased inflammation and intestinal microbial imbalance could interact to cause intestinal dysfunction and sympathetic nervous system dysfunction, which could accelerate the progression of hypertension [43]. Studies using fecal microbiota transplantation and bacterial genome analysis have found that abnormal richness of the gut microbiota is significantly associated with hypertension [44]. The diversity of the gut microbiota was drastically reduced in hypertensive patients and animal models. The structure and function of microorganisms were disrupted, leading to a change in fermentation products. Zheng et al. showed that a high-salt diet inhibits the growth of beneficial bacteria, such as *Bifidobacteria*, in the intestinal mucosa, thereby reducing intestinal mucosal homeostasis [45]. Based on the characteristics of the microbiome and microbial metabolites, it is possible to effectively distinguish between hypertensive patients and individuals who have not undergone drug treatment. This method can also serve as a potential means of preliminary identification of populations with hypertensive symptoms. Changes in the gut microbiota under a high-salt dietary pattern are shown in Table 2.

## 5. Intestinal Barrier Mechanisms of High-Salt-Diet-Induced Hypertension

The intestinal barrier consists of mechanical, chemical, microbial, and immune barriers, which are closely linked to the gut microbiota. A high-salt diet could disrupt homeostasis in the gut, with effects on gut microbiota, intestinal metabolism, and intestinal immunity. The gut microbiota of healthy individuals is usually in balance, participating in the metabolism of food and synthesizing metabolites that are either beneficial or harmful to the body. However, a high-salt diet disrupts this balance and triggers metabolic disorders, resulting in altered levels of microbiota-related metabolites. For instance, it could increase the accumulation of toxic metabolites in the intestine, promoting the passage of these toxic substances through the intestinal barrier to other tissues and organs, thereby increasing the burden on the body [49]. In recent years, more and more studies have shown that the occurrence of hypertension is closely related to the gut microbiota and that a disturbed intestinal microenvironment affects the integrity of the intestinal barrier by altering the composition of the gut microbiota and its associated pathways [20]. Metabolites of gut microbiota could stimulate beneficial or harmful immune responses, participate in inflammatory responses, and play an important role in the mechanisms of hypertension development.

### 5.1. Disruption of the Intestinal Barrier-Induced Hypertension

#### 5.1.1. Disruption of the Intestinal Mechanical Barrier

**Figure 2:**
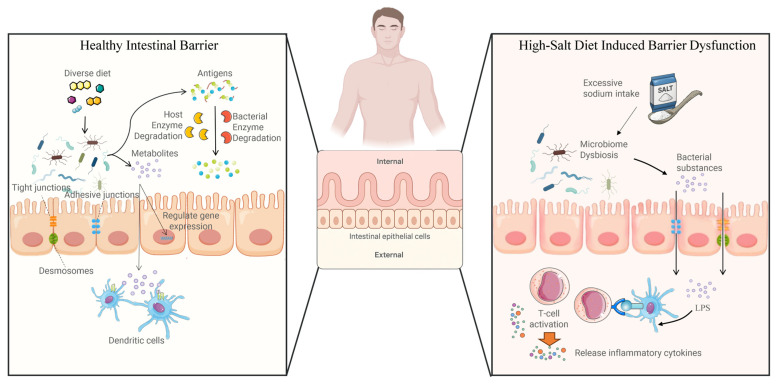
The mechanism by which excessive salt intake disrupts the mechanical barrier of the intestinal tract.

The mechanical barrier is the primary component of the intestinal barrier, consisting of intestinal epithelial cells and intercellular connections that prevent pathogenic microorganisms and harmful substances from entering the bloodstream. The mucus layer forms the outermost layer of the intestinal barrier. It protects epithelial cells and creates a local ecological environment for the gut microbiota. Mucoproteins were the primary components of the mucous layer, and they were specifically responsible for the binding and degradation of mucopolysaccharides [70]. Studies have shown that there was a tight balance between mucin production and degradation under healthy conditions, but under a high-salt dietary pattern, dysregulated gut ecology might lead to degradation of the mucus layer and eventual destruction of the intestinal epithelial layer tissue [71]. Tight junctions and adherens junctions are both protein complexes. Tight junctions seal the apical gaps between intestinal epithelial cells and maintain homeostasis by regulating the diffusion of ions, solutes, and microorganisms across the intestinal barrier [72]. Components of tight junction proteins include claudin, occludin, tricellulin, junctional adhesion molecule-A, zonula occludens, and cingulin [20]. The expression level of tight junction proteins directly reflects the integrity of the intestinal barrier. The claudin proteins in the gastrointestinal tract are mainly claudin-2 and claudin-15, which allow Na^+^ to be transported via the paracellular pathway [73,74]. IL-6 and IL-13 have been shown to alter the expression of claudin-2 protein [75]. Studies on mice lacking claudin-15 revealed that their sodium permeability was reduced [76]. The intestine absorbs the majority of dietary sodium ions. A high-salt diet promotes the production of claudin-2 protein, which increases sodium ion absorption, exacerbating inflammation and raising blood pressure [77]. Damage to the intestinal barrier increases the risk of gut microbiota and other harmful substances entering the body’s internal environment. For example, the accumulation of IsoLG-protein adducts promotes hypertension and inflammation; the LPS-induced MAPK/NF-κB pathway has also been demonstrated to be associated with the development of hypertension and inflammation. Figure 2 shows the mechanism by which excessive sodium intake causes gut microbiota imbalance and disrupts the intestinal barrier. Although current research has not yet proposed effective methods for repairing intestinal tight junctions, nor are there any FDA-approved drugs for treating epithelial barrier dysfunction, dietary regulation may be an effective way to maintain and protect intestinal permeability as a bodily function damaged by diet, thereby alleviating hypertension.

#### 5.1.2. Disruption of the Intestinal Chemical Barrier

Chemical barriers are an important part of the intestinal defense mechanism and include various chemical substances such as digestive juices, digestive enzymes, lysozyme, bile acids, mucopolysaccharides, glycoproteins, glycolipids, and short-chain fatty acids (SCFAs) [78]. The mucus layer provides a local ecological environment for the gut microbiota. It can bind and break down the mucus layer, playing an important regulatory role in the chemical barrier. It may be damaged due to a high-salt diet [71]. Bile acids regulated by the gut microbiota, which are affected by a high-salt diet, can destroy or bind bacterial toxins in the intestine, forming complexes that are difficult to absorb, thereby inhibiting bacterial translocation and endotoxin absorption [79]. They influence the intestinal barrier, metabolic pathways, and vascular tension to control blood pressure. For instance, the G protein-coupled bile acid receptor 1 promotes vasodilation of vascular endothelial cells and lowers blood pressure by inhibiting endothelin release and regulating NO synthesis [80]. The gut microbiota can metabolize indigestible proteins and carbohydrates into SCFAs, such as acetic acid, propionic acid, and butyric acid. These SCFAs can be absorbed by the distal intestine, providing energy to intestinal epithelial cells and thereby enhancing intestinal barrier function. Lactic acid-producing bacteria in the gut microbiota prevent salt-induced T cell activation and hypertension [57]. Because of the presence butyrate, the intestinal mucosa has deliberately adapted to use different oxygen levels, supporting mucosal homeostasis. Butyrate is used by intestinal epithelial cells for a variety of purposes that support mucosal homeostasi [81]. The amount of SCFAs in feces and the makeup of the gut microbiota are both impacted when rats are fed salt [69]. Fecal samples from rats with hypertension induced by a high-salt diet showed notably lower levels of butyrate, propionate, and acetate [82]. A high-salt diet downregulates γ-aminobutyric acid (GABA) expression in the rat intestine [83]. GABA participates in the regulation of the gut microbiota and key biomarkers, exhibiting close associations with disease. It may function as an intestinal mediator by participating in enteric nervous system circuits to regulate gastrointestinal function. Targeting excitatory GABA (A) or inhibitory GABA (B) receptors can modulate intestinal mucosal function [84]. It is evident that most intestinal chemicals originate from metabolites produced by the gut microbiota. Compounds such as bile acids, SCFAs, and GABA help maintain intestinal barrier function and promote health. However, a high-salt diet reduces the levels of these compounds and increases the production of pro-inflammatory factors, further compromising the chemical barrier function of the intestine and exacerbating the development of hypertension.

#### 5.1.3. Disruption of the Intestinal Biological Barrier Induced Hypertension

The gut microbiota participated in the onset and development of hypertension through multiple mechanisms. Probiotics and pathogenic bacteria in the intestine constrain each other, achieving a state of balance and forming a biological barrier on the intestinal mucosa. The structure of the bacterial community on the intestinal mucosa and its metabolic products are key factors influencing the function of the intestinal barrier and the development of hypertension. Its gene expression plays an important regulatory role in intestinal nutrient absorption and the human immune system, and is closely related to the onset and development of hypertension [85,86]. The gut microbiota affects intestinal barrier function directly or indirectly. Animal studies indicated that, in mice, long-term high-salt diets significantly reduced the abundance of Bacilli, Lactobacillales, Leuconostocaceae, and *Leuconostoc* in the intestines of mice [41]. Compared with normal Wistar–Kyoto rats, spontaneous hypertensive rat models showed significantly reduced gut microbial diversity and abundance. After four weeks of high-salt diet intervention, the α diversity of the gut microbiota of rats was significantly reduced, with a significant decrease in the abundance of 22 genera and a significant increase in the abundance of nine genera. The increased bacteria mainly belonged to the phyla Actinobacteria, Firmicutes, and Proteobacteria [41,46]. Studies on hypertensive patients indicated that nine bacterial genera, including *Bulleidia, Clostridium*, *Mogibacteriaceae*, *Enhydrobacter*, *Pseudidiomarina*, *Novosphingobium*, *Chryseobacterium*, *Catenibacterium*, and *Klebsiella*, significantly increased in abundance under a high-salt diet, and these changes were positively correlated with sodium intake [60]. *Lactobacillus* and *Bacteroides* were significantly negatively correlated with the incidence of hypertension [46]. *Klebsiella* was more abundant in hypertensive intestines. A high-salt diet promoted the colonization of *Klebsiella* by inhibiting gastric acid secretion, and the increased *Klebsiella* affected the intestinal immune system, promoting the occurrence of hypertension. Fecal transplant experiments indicated that the feces of hypertensive patients could cause an increase in blood pressure in germ-free mice [60]. In summary, when the intestinal microbial barrier function was normal, it could effectively block the entry of harmful substances such as bacteria, viruses, and toxins, thereby reducing vascular damage and inflammatory responses, which was conducive to maintaining the homeostasis of the intestinal environment. Once the composition of the gut microbiota changes, this imbalance can increase the permeability of intestinal epithelial cells due to differences in the adhesion abilities of different bacterial species, inducing a shift in the microbiota and allowing pathogenic bacteria and their toxins in the intestine to enter the bloodstream. It triggers a systemic inflammatory response, leading to damage to the vascular endothelium and vascular sclerosis, ultimately leading to elevated blood pressure and the development of hypertension [87].

### 5.2. Accumulation of Intestinal IsoLG-Protein Adducts Induces Hypertension

**Figure 3:**
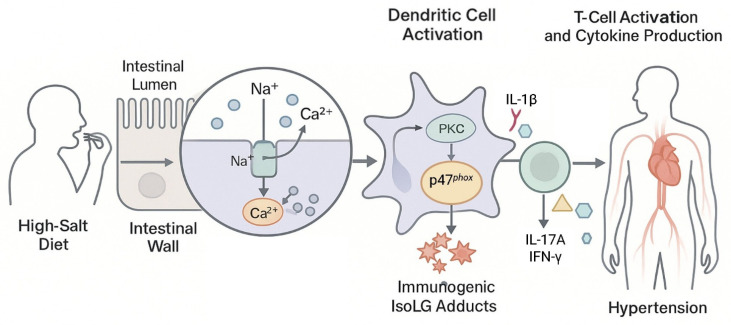
Excessive sodium intake accumulated IsoLG-protein adducts and induced hypertension.

DCs play a key role in the adaptive immune response of the intestine under a high-salt diet, a process mainly due to the accumulation of immunogenic IsoLGs [88]. IsoLGs are byproducts of lipid oxidation that can be altered by rapidly reacting with their own Lys. Modified IsoLGs prevent DCs from activating T cells, thereby lowering the risk of hypertension [89]. In addition, the infiltrating T cells around the kidneys and blood vessels respond to the stimulation of hypertension, release inflammatory factors, and thereby induce functional disorders of the kidneys and blood vessels, further accelerating the development of hypertension [90,91]. However, researchers have discovered an interesting opposite phenomenon in the high-salt hypertension animal model. In experiments on salt-sensitive hypertensive mice, IsoLGs reversed activated DCs, causing immune cell activation, which exacerbated inflammation and worsened hypertension [92,93,94]. In mice fed a high-salt diet, there was an increase in the infiltration of inflammatory cells (such as CD11c^+^, CD45^+^, CD3^+^, CD4^+^, and CD8^+^) in the intestine and a significant accumulation of IsoLG-protein adducts [88]. In a gut microbiota analysis of salt-sensitive hypertensive mice, a reduction in the abundance of lactic acid-producing probiotics was observed. Lactic acid-producing probiotics could prevent salt-induced T cell activation [60]. Thickening and fibrosis of small artery walls were observed in patients with hypertension, along with increased infiltration of immune cells in the intestinal wall and a significant increase in the accumulation of IsoLGs in colon sections [88]. By triggering protective immune responses against infections and promoting immune tolerance to innocuous antigens, DCs normally preserve intestinal homeostasis. However, gut microbiota dysbiosis impairs this function under high-salt dietary conditions, which ultimately results in immunological imbalance. A high-salt diet disrupts the intestinal microbial barrier, which increases the presentation of immunogenic IsoLG-protein adducts in DC11c^+^ antigen-presenting cells, ultimately leading to T cell activation and promoting the production of IFN-γ, a cytokine associated with hypertension. The intestinal mucosa is rich in Na^+^ transporters. When the extracellular Na^+^ concentration increase, DCs sense this change through amiloride-sensitive transporter, causing calcium to enter the cells through Na^+^/Ca^2+^ exchanger. Creating an environment in which intracellular calcium concentrations rise, activating protein kinase C (PKC) [95]. PKC phosphorylation activates the nicotinamide adenine dinucleotide phosphate (NADPH) oxidase subunit p47*^phox^*, leading to the production of superoxide and the formation of immunogenic IsoLG-protein adducts, which activate DCs [88]. Activated DCs release IL-1β, which further stimulates T cells to produce cytokines IL-17A and IFN-γ and accelerates the development of hypertension [88]. When these DCs, activated by immunogenic IsoLG-protein adducts, were transplanted into healthy mice and injected with a hypotensive dose of AngII, the mice developed hypertension [95]. Based on available evidence, we hypothesized that excessive dietary sodium would be directly absorbed by cells via the Na^+^/Ca^2+^ exchanger, resulting in immunogenic IsoLG-protein adducts that activated DCs and T cells and secreted cytokines, disrupting the intestinal microbial barrier through positive feedback and exacerbating hypertension. In summary, excessive dietary sodium intake can cause hypertension via the interaction of immunogenic IsoLG-protein adducts and gut microbiota (Figure 3).

### 5.3. Promoting LPS Activation of the MAPK/NF-κB Pathway Induces Hypertension

**Figure 4:**
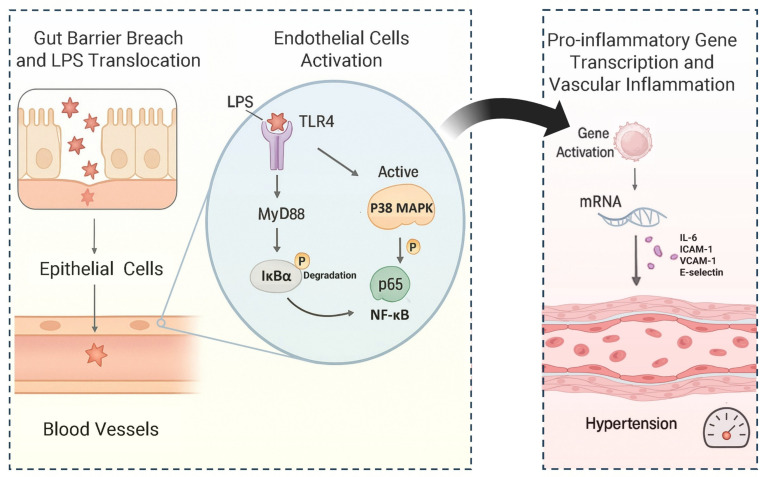
LPS translocation leads to hypertension.

LPS is the main component of the outer membrane of the cell wall of Gram-negative bacteria. In animal experiments, it was a common intracellular toxin. It usually serves as a typical pathogen molecular pattern component for the study of Gram-negative bacteria, stimulating and inducing vascular dysfunction. It is also a commonly used stimulant for studying myeloid differentiation primary response 88 (MyD88)-dependent inflammatory pathways. For example, under conditions of hypertension and a high-salt diet, the abundance of *Klebsiella* significantly increased. The cell wall LPS of these *Klebsiella* could stimulate the body’s immune system, thereby promoting the development of intestinal inflammation [49]. Microbial dysbiosis leads to impaired intestinal barrier integrity and tight junction protein expression, allowing LPS to freely enter the bloodstream [96]. MyD88 is a crucial junctional protein that plays a significant role in the inflammatory pathway of hypertension. The signals it mediated mainly activate MAPK (ERK, JNK, and p38) and transcription factor NF-κB, driving the expression of pro-inflammatory cytokines and participating in the vascular lesions of hypertension. In human umbilical vein endothelial cells, after 30 min of stimulation with 100 ng/mL LPS, the level of nuclear factor κB inhibitor (IκBα) α in the cytoplasm was significantly reduced, leading to increased NF-κB activity [96]. Activated p38MAPK promoted the phosphorylation of IκB, causing IκB to dissociate from NF-κB, leading to the activation of NF-κB and its nuclear translocation. However, there were differences in research findings regarding the relationship between p38MAPK and NF-κB in the MyD88 pathway. According to the literature, when the selective p38MAPK inhibitor (SB203580) was pre-treated at a concentration of 25 μM for 1 h, it did not significantly alter the level of NF-κB, suggesting that these two signaling pathways might be independent in this model [96]. However, Li et al. conducted a study that revealed that the same dose of p38MAPK inhibitor could partially prevent the translocation of NF-κB, and another p38MAPK inhibitor (SB202190) also yielded similar results [97,98]. These findings suggested that in human umbilical vein endothelial cells stimulated by LPS, p38MAPK might act as an upstream regulatory factor of NF-κB and influence blood pressure. LPS levels in hypertensive patients were significantly higher than in normal individuals [76]. Experiments with SHR rats showed that blocking TLR4 significantly reduced p38MAPK activity, NF-κB phosphorylation, and IL-6 protein expression in rats [99]. Intraperitoneal injection of 10 μg/10 g body weight of LPS significantly increased NF-κB activity in wild-type mice, but TLR4 knockout mice treated with the same dose did not show this response [100]. These results reveal that LPS-induced vascular inflammation depends on TLR4 activation. The application of the p38MAPK inhibitor SB239063 could eliminate the increase in blood pressure caused by a high-fat and high-salt diet [101]. The mechanism by which LPS caused hypertension is shown in Figure 4. In short, high-salt diets cause ecological imbalances that damage the intestinal barrier, allowing LPS to cross into the bloodstream and activate the inflammatory pathway mediated by TLR4, ultimately triggering a systemic inflammatory disease mechanism including hypertension. This mechanism involves p38MAPK activation, IκB degradation, and p65 NF-κB nuclear translocation. This upregulates the transcription of inflammatory factors (such as IL-6) and adhesion molecules (such as ICAM-1, VCAM-1, and E-selectin), leading to the development of vascular inflammation and hypertension.

## 6. Dietary Therapy for Hypertension

The treatment of hypertension usually relies on drug intervention, but drugs for treating hypertension need to be taken regularly and have side effects. As described in the latest review on antihypertensive drug therapy, although combination therapy is more effective at lowering blood pressure than simply increasing the dose of a single drug, and combination formulations can reduce medication burden, patients’ long-term treatment costs and adherence to regular medication schedules may lead to therapeutic inertia over time [102]. Non-pharmacological treatment of hypertension has become a widely adopted approach to managing hypertension in both developed and developing countries and is an important component of hypertension management, aiming to manage blood pressure through non-pharmacological interventions that modify dietary patterns. While the effects may not be as immediate as those of medication, this approach offers patients a broader range of options. Dietary therapy based on evidence-based medicine has become a safe, economical, and practical sustainable intervention model for hypertension. Research showed that dietary therapy could not only effectively alleviate psychological stress in patients with hypertension but also enhance the efficacy of antihypertensive drugs, providing significant benefits for the treatment of hypertension [103].

### 6.1. Supplement with Natural Blood Pressure-Lowering Food Resources

The classic masterpiece of traditional Chinese medicine (TCM) called *Su-wen: Zangqi Fashi Lun* mentioned that the principles of food therapy combinations were “five grains as nourishment, five fruits as supplement, five meats as benefit, five vegetables as addition” [104]. It reflects the significance of a diverse diet for human health. Many natural food resources contain effective components with the function of lowering blood pressure, such as polyphenols, dietary fiber, GABA, and omega-3 fatty acids, and so on. Adjusting the dietary structure and increasing the intake of food resources with blood pressure-lowering effects in the diet could play a significant role in the prevention and supplementary treatment of hypertension.

#### 6.1.1. Supplement with Plant-Based Foods

Plant-based foods such as fruits and vegetables in the diet can provide the body with a rich variety of phytochemicals, including anthocyanins, quercetin, resveratrol, epigallocatechin gallate, curcumin, carotenoids, organosulfur compounds, phytoestrogens, and so on. These phytochemicals improved intestinal barrier function by promoting the proliferation of beneficial gut microbiota, inhibiting the expression of pro-inflammatory factors, and promoting the expression of anti-inflammatory factors, thereby adjusting the intestinal barrier function and ultimately achieving the goal of lowering blood pressure through the “brain-gut” axis [20]. Phytochemicals can also reduce NF-κB signaling pathway transmission, inhibit the expression of transcription activator (STAT), myosin light chain kinase (MLCK), and mitogen-activated protein kinase, and enhance antioxidant activity, thereby alleviating intestinal inflammatory manifestations [105]. Polyphenols are abundant in plant-based foods such as fruits and vegetables. They are the most frequently consumed and most abundant type of antioxidant and possess anti-aging and anti-inflammatory properties, manifested as limiting or preventing oxidative damage to cellular components, DNA, proteins, and fats associated with aging-related diseases; alleviating vascular oxidative stress; and reducing intercellular cell adhesion molecule-1, vascular endothelial adhesion molecule-1, monocyte chemoattractant protein-1, TNFα, IFN-γ, IL-6, IL-8, and NF-κB, which can alleviate hypertension [106,107]. When blackcurrant polyphenols were administered orally to spontaneously hypertensive rats, they exhibited a blood pressure-lowering effect similar to that of the antihypertensive drug captopril (at a dose of 10 mg/kg) within 4–8 h [108]. Pomegranate polyphenols could significantly lower blood pressure in spontaneously hypertensive rats, and the mechanism might be related to the regulation of vasoactive substances such as angiotensin II, endothelin, and nitric oxide expression [109]. Blueberry leaf polyphenols could effectively inhibit angiotensin-converting enzyme activity and significantly reduce systolic and diastolic blood pressure in spontaneously hypertensive rats [110]. Polyphenols could also regulate the gut microbiota, stimulate the proliferation of probiotics like *Lactobacillus* and *Bifidobacterium*, and inhibit the growth of bacteria like *Helicobacter pylori* and *Staphylococcus aureus*, all of which helped to relieve hypertension [111]. Changing our diet and consciously consuming foods high in phytochemicals like anthocyanins, quercetin, and resveratrol can help lower blood pressure.

#### 6.1.2. Supplement with PFAs

Foods such as fish, nuts, and vegetable oils supplement the human body with PFAs. PFAs have more than one double bond structure, such as omega-3 fatty acids and omega-6 fatty acids, which are mainly found in eicosapentaenoic acid and docosapentaenoic acid. A diet rich in omega-3 fatty acids can enhance the bioavailability of nitric oxide and promote blood pressure reduction by mediating vasodilation through metabolites such as epoxides and eicosanoids [112,113]. PFAs promote the abundance of beneficial bacteria such as *Bifidobacterium* and *Lactobacillus* in the intestines and stimulate the release of SCFAs [114]. Research showed that these products inhibit the release of inflammatory factors such as TNF-α, IL-1β, and IL-6 and produce lipid mediators to achieve anti-inflammatory effects, thereby lowering blood pressure [115]. PFAs have a clear regulatory effect on blood pressure [116]. We suggest that people with high blood pressure can consciously choose oils with high content of PFAs as the preferred choice for cooking or direct use. By consuming them in a slow, small but continuous manner, the effect of regulating blood pressure can be achieved.

#### 6.1.3. Supplement with Probiotics and Prebiotics

According to the definition of the Food and Agriculture Organization of the United Nations and the World Health Organization, probiotics are *“live microorganisms, when administered in adequate amounts, confer a health benefit on the host”.* Nowadays, the most popular probiotic mixtures are lactate-producing *Bifidobacterium* and *Lactobacillus*. The functions of probiotics include increasing microbial diversity; competing with pathogens; producing bacteriocins, vitamins, and SCFAs; enhancing immunity in the intestine; increasing the production of anti-inflammatory cytokines such as IL-10; reducing intestinal permeability and oxidative stress; producing neurotransmitters; and improving mucosal integrity and barrier function [112,117]. Foods rich in probiotics include yogurt, sauerkraut, fermented black soybean, natto, soy sauce, and cheese [118,119]. Prebiotics are polysaccharides found in natural foods that are difficult for human enzymes to digest, but can be utilized by probiotics. These include fructooligosaccharides, galactooligosaccharides, isomaltulose oligosaccharides, xylooligosaccharides, arabinooligosaccharides, pectin oligosaccharides, and resistant starch, which are often fermented by the gut microbiota in the colon [20]. Supplementing with prebiotics could specifically promote the proliferation of beneficial bacteria in the gut, thereby increasing SCFA levels while reducing LPS and IL-6 content, ultimately promoting overall health [120]. Foods rich in prebiotics include artichokes, onions, garlic, leeks, soybeans, chicory root, honey, bananas, plant seeds, and fiber [121]. Fermented foods are rich in probiotics and prebiotics. The vitamins, milk peptides, bacteriocins, and immune enhancers produced during the fermentation process have been shown to play a positive role in balancing intestinal permeability and improving barrier function [118,122]. The combined use of probiotics and prebiotics has positive implications for damaged intestinal barriers or the prevention of intestinal barrier damage.

**Figure 5:**
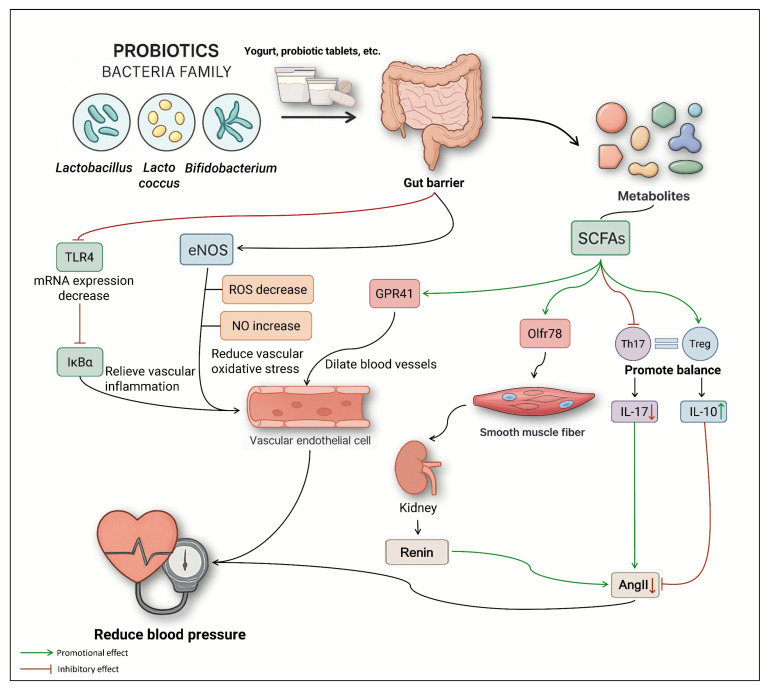
Different regulatory pathways of probiotics on hypertension.

Studies have shown that the use of probiotics could significantly lower blood pressure [123]. Probiotic adjunctive therapy in hypertension has the potential to produce SCFAs, improve vascular oxidative stress, repair endothelial cell dysfunction, and reduce vascular inflammation [124]. Hypertension is closely related to oxidative stress. When blood vessels are damaged, their impaired contraction and relaxation functions are the direct cause of abnormal blood pressure [125]. Nitric oxide synthase in blood vessels synthesizes NO, which inhibits the production of reactive oxygen species and protects the vascular endothelium. Probiotics can maintain blood pressure levels by restoring the balance of reactive oxygen species and NO in blood vessels. Kefir had antioxidant and antihypertensive properties [126]. Directly feeding SHR mice with *Lactobacillus fermentum* CECT5716 and *Bifidobacterium brevis* CECT7263 significantly increased the number of butyrate-producing bacteria in the mouse intestine and effectively prevented an increase in blood pressure [127]. Probiotics regulate hypertension via four pathways: (1) LPS/TLR4, (2) GPR41/Olfr78, (3) Th17/Treg, and (4) eNOS/NO (Figure 5). Vascular endothelial cell (VEC) dysfunction is a key factor in the development of hypertension, which exacerbates VEC dysfunction, resulting in a vicious cycle [124]. Long-term intake of propionate can alleviate endothelial dysfunction. Supplementing with *Lactobacillus plantarum* could improve endothelial dysfunction and reduce systemic inflammation, thereby inhibiting blood pressure fluctuation [127,128]. Transplantation of *Lactobacillus murinus* can prevent the occurrence of salt-sensitive hypertension [129]. *Lactobacilli* taken orally can cause the formation of tolerant dendritic cells, reducing the risk of atherosclerosis [60]. Overall, these findings reinforce the importance of the gut microbiota in the treatment of cardiovascular disease. The close relationship between gut microbiota and normal intestinal barrier function, and its key role in regulating intestinal balance. By supplementing the microbial species (probiotics) that are inhibited by a high-salt diet, it is expected that the high-salt-induced hypertension can be alleviated by repairing the intestinal barrier.

#### 6.1.4. Supplement with FMHS

FMHS referred to substances that are both TCM and food. TCM theory has long held the concept that “food and medicine have the same origin”. In recent years, there has been a structural shift in the global spectrum of chronic noncommunicable diseases and a significant increase in public health management awareness. The promulgation of the *Chinese Catalog of Dual-Use Food-Medicinal Substances* and the development of functional foods have further promoted the feasibility of FMHS as a daily dietary supplement. The application value of FMHS in the field of hypertension prevention and control has attracted widespread attention. FMHS relies on its natural properties, low toxicity, and multi-target regulatory advantages to intervene in vascular homeostasis through multiple pathways. As shown in Table 3, FMHS such as *Mori folium*, *Semen raphani*, *Crataegi fructus*, and *Puerariae lobatae radix*, etc., have potential blood pressure-lowering effects. *Mori folium* extract lowered blood pressure by increasing endothelial-dependent vasodilation and NO bioactivity [130]. The water-soluble alkaloids in *Semen raphani* regulated blood pressure by activating the NOS system to mediate peripheral resistance control [131]. *Crataegi fructus* flavonoids and kudzu isoflavones achieve synergistic blood pressure reduction by regulating the metabolic balance of endothelin-1 and Ang II [132,133]. FMHS is frequently used as a medicinal additive in TCM formulations or as an active ingredient in functional foods. However, its use still necessitates a balance of efficacy and safety. Exploring the combination of FMHS and drugs is the future direction for treating hypertension and other diseases. However, we firmly believe that supplementary substances derived from FMHS and related products will become an important part of the dietary therapy for hypertension.

#### 6.1.5. Supplementation with Minerals and Vitamins

Minerals and vitamins play an important role in shaping the intestinal barrier. Vitamins maintain intestinal homeostasis by repairing the mechanical barrier of damaged intestines. They can enhance intestinal tight junction proteins to maintain intestinal permeability. Studies have shown that vitamin A could increase the relative expression levels of ZO-1, claudin-1, and occludin in the intestine and could also prevent inflammation in the intestine. Vitamin D increases the relative expression levels of ZO-1, claudin-1, claudin-2, and E-cadherin in the intestine, which have a long-term effect on immune regulatory function [142,143]. Minerals influence the physiological activities of the gut microbiota, regulating tight junction protein activity and intestinal permeability [144]. Although studies have shown that minerals and vitamins have a significant role in repairing the intestinal barrier, there is a lack of research on the effect of their supplementation on lowering blood pressure in patients with hypertension. Further research is required in this area. 

### 6.2. Changing Dietary Patterns

In the case of hypertension caused by a high-salt diet, lowering salt intake and optimizing dietary structure are effective treatment strategies for repairing the damaged intestinal barrier. Dietary patterns, as a controllable factor, have a significant impact on individual health, and individuals can play a positive or negative role. Therefore, dietary management is expected to play an important role in the development of personalized medicine. DASH, MD, and KD are common dietary patterns used to prevent and treat hypertension.

#### 6.2.1. DASH

DASH originated and developed in the late 20th century as part of a large-scale hypertension prevention program at the National Institutes of Health in the United States. They found that SBP decreased by approximately 6–11 mmHg in subjects who followed this dietary pattern alone [145]. The DASH diet encourages the consumption of fruits, vegetables, lean meats, low-fat dairy products, whole grains, poultry, fish, and nuts, while restricting red meat, sweets, and sugary beverages. It also encourages people to consume less sodium, saturated fat, total fat, and cholesterol while increasing their intake of potassium, calcium, and magnesium. DASH helps to lower blood sugar, triglycerides, low-density lipoprotein cholesterol, and insulin resistance, and is an auxiliary treatment option for various metabolic syndromes [145]. In a meta-analysis, reduced sodium intake and increased potassium intake were identified as the most important mechanisms of the DASH diet’s antihypertensive effects [146]. Insulin resistance is often accompanied by compensatory hyperinsulinemia, which causes oxidative stress in blood vessels and contributes to hypertension [147]. Potassium can increase urinary sodium excretion, reduce insulin resistance, mitigate oxidative damage, and is of great significance for the treatment of salt-sensitive hypertension patients [148]. Potassium can also reduce the contraction of vascular smooth muscle to lower blood pressure [149]. The DASH diet can improve the intestinal barrier function in hypertensive patients. After following the DASH diet for two weeks, zonulin levels in the blood of 20 hypertensive patients not taking antihypertensive medication were significantly reduced [150]. A study involving 20 patients with hypertension showed that those who adopted the DASH diet had significantly lower systolic and diastolic blood pressure compared to those on a regular diet. The DASH diet reduced blood pressure by improving and upregulating the bioavailability of nitric oxide, promoting early diuresis, and reducing oxidative stress levels [151]. The composition of DASH is consistent with the aforementioned substances in this article that are rich in polyphenols, quercetin, etc. and have blood pressure-lowering effects. The mechanism by which DASH lowered blood pressure may involve improving the availability of nitric oxide, promoting urination, reducing oxidative levels, restoring intestinal barrier function, etc. In summary, DASH is an effective dietary intervention method for patients with hypertension, and it also has significant importance for preventing hypertension in normal individuals.

#### 6.2.2. MD

MD includes high-fiber and low-gluten foods, fish high in Omega-3 fatty acids, polyphenol-rich fruits and vegetables, and reduces the intake of animal fats, processed meats, high-salt, and high-gluten diets. A randomized controlled meta-analysis showed that both hypertensive patients and healthy adults who received an MD dietary intervention experienced a significant reduction in blood pressure [152]. The blood pressure-lowering effect was particularly pronounced in participants with higher baseline systolic blood pressure and longer follow-up periods. MD is also a widely used nutritional intervention for obesity management, and its positive effect on lowering blood pressure might be a long-term result of weight loss. The main source of fat in MD was extra-virgin olive oil (EVO). EVO improves intestinal permeability and inhibits the NF-κB signaling pathway, lowering levels of pro-inflammatory factors like IL-1β, TGFβ, and IL-6 and reducing chronic inflammation. In addition, MD was associated with increased SCFAs in the gut, improved microbial diversity and stability, as well as an increase in the abundance of lactic acid-producing bacteria. It also raises the levels of IL-10 and IL-22, and lowers IL-17, thereby enhancing immune function [111]. Research has also indicated that MD is rich in various plant-based chemicals. These components can promote the growth of beneficial bacteria in the intestines, reduce the production of inflammatory factors, and increase the expression of anti-inflammatory factors, thereby improving the function of the intestinal barrier [20]. The composition of MD’s food and its mechanism of lowering blood pressure are also consistent with the dietary supplementation of polyphenols and PFAs mentioned in this article, which assist in lowering blood pressure. MD dietary polyphenols can deregulate NADPH oxidase and NF-κB-mediated oxidative stress and metabolic inflammation [153]. Dietary fiber, EVO, and PFAs in MD can regulate the gut microbiota, promote the proliferation of beneficial bacteria such as *Lactobacillus* and *Bifidobacterium*, inhibit the growth of *Helicobacter pylori* and *Staphylococcus aureus*, and enhance the intestinal barrier function to achieve blood pressure regulation [111]. As a result, we believe that MD should have a greater impact on salt-sensitive hypertensive patients following intestinal barrier disruption.

#### 6.2.3. KD

KD was first established in the early 20th century for drug-resistant epilepsy, especially in children. KD is a dietary pattern that is high in fat, contains sufficient protein, and has extremely low levels of carbohydrates. The ratio of fat to protein and carbohydrates is 3:1 or 4:1, with ketone bodies as the main source of energy. KD has been proven to be an effective intervention for treating metabolic syndrome, but the specific mechanisms by which it affects blood pressure remain unclear [154]. The survey showed that the number of participants whose blood pressure was controlled by only following the KD was significantly lower than that of those who followed the KD and engaged in physical exercise simultaneously. Meanwhile, intervention measures for controlling weight and reducing body fat rate would improve the blood pressure condition of each participant [155]. After 45 days on a very low-calorie KD trial, 137 obese women between the ages of 35 and 55 found a reduction in SBP of 12.89% and a reduction in DBP of 10.77% and the participants also lost weight [156]. After eight weeks on an extremely low-carbohydrate ketogenic diet, fermentative dysbiosis was observed. At the conclusion of the dietary intervention, patients’ urinary indican concentrations increased significantly (42.00 mg/L); a significant increase in urinary concentrations occurred (57.50 mg/L). Intestinal translocation of bacteria was also observed in patients, with serum LPS concentrations (0.046 ng/mL) significantly higher than at baseline (0.028 ng/mL). Interestingly, circulating levels of TNF-α were significantly reduced at the end of treatment, while circulating levels of the anti-inflammatory cytokine IL-10 were significantly elevated at the conclusion of the therapy [157]. The primary effect of KD on hypertension is weight control, particularly for obese patients with high body fat. The development of long-term inflammation is a characteristic of obesity. Increased reactive oxygen species in patients’ blood vessels led to oxidative stress and ultimately endothelial dysfunction. Compared to KD mice, those administered butyrate exhibited significantly enhanced intestinal barrier function, along with markedly reduced bacterial translocation, systemic inflammatory response, and mortality [158]. The high-sensitivity C-reactive protein levels of subjects who accepted the very low-calorie KD significantly decreased [156]. After correlating this with systolic and diastolic blood pressure, it was found that the reduction in inflammatory factors was the main factor contributing to the lowering of blood pressure in the KD dietary pattern. In addition, long-term chronic inflammation reduced the bioavailability of NO, interrupted the main function of vasodilators, and affected normal vascular dilation [159]. Long-term use of KD might induce certain adverse reactions, including constipation and hypercholesterolemia. While the KD diet better meets the blood pressure reduction needs of obese patients, its effects on gut microbiota composition and function, as well as intestinal barrier integrity, may pose potential risks. Different fat sources in KD diets exert varying impacts on the intestinal barrier. In a series of studies on mice, it was found that high levels of the omega-6 fatty acid linoleic acid may render the intestinal barrier more susceptible to damage, whereas saturated fats—particularly medium-chain triglycerides—appear to pose less harm [160,161]. Furthermore, a ketogenic diet rich in saturated fats promotes the expression of tight junction proteins in the colon [162]. Therefore, KD may be more suitable for patients with obesity and hypertension. Although KD has been proven to have the effect of lowering blood pressure, its mechanism of doing so is related to the reduction in inflammatory factors in the body. However, it might damage the intestinal barrier or cause metabolic disorders. Therefore, it should be used under the guidance of professionals such as doctors and nutritionists, combined with one’s own condition.

In conclusion, the food composition of the above three dietary patterns is consistent with the supplementary natural food resources mentioned above in this article. For example, the main dietary composition in DASH includes fruits and vegetables rich in polyphenols, quercetin, resveratrol, etc., while in MD, olive oil rich in PFAs is used. These three diets can be chosen as dietary options for patients with hypertension. Changing from a high-salt, high-fat diet pattern to a DASH, MD, or KD diet pattern can effectively assist in lowering blood pressure. However, they also have different applicability for different populations. For example, KD may be more suitable for patients with hypertension and obesity. Future research should involve designing different diets for patients with hypertension caused by different factors, in order to achieve precise nutrition in the dietary therapy for hypertension, assist in lowering blood pressure, and alleviate the social and economic burdens caused by hypertension.

## 7. Conclusions

The development of society and changes in dietary habits have led to excessive salt intake far exceeding the recommended amount. At the same time, combined with lifestyle changes, the global incidence of hypertension has continued to rise and is showing a trend of affecting younger individuals. However, contrary to its high incidence rate, less than 50% of hypertensive patients have their blood pressure effectively controlled due to economic conditions or regular medication. A high-salt diet can directly cause high blood pressure and also promote the onset and development of hypertension by altering the gut microbiota and damaging the intestinal barrier. The intestinal mucosal barrier is the body’s first line of defense and the main site of sodium absorption from the diet. A long-term high-salt diet can damage the mechanical, chemical, and microbial barriers of the intestine, causing bacteria attached to the intestinal mucosal barrier to become displaced. Bacteria, viruses, and intestinal toxins penetrate into the bloodstream, causing damage to the vascular endothelium and vascular hardening, which leads to high blood pressure. Furthermore, excessive dietary sodium intake could also induce hypertension through the interaction between immunogenic IsoLG-protein adducts and intestinal microorganisms, or by triggering intestinal inflammation, stimulating the immune system, and activating the MAPK/NF-κB pathway to cause hypertension.

In the prevention and treatment of hypertension, dietary therapy is a sustainable intervention model for hypertension that is supported by evidence-based medicine. Supplements and foods rich in specific nutrients have a significant effect on regulating blood pressure. These include natural antihypertensive ingredients (polyphenols, quercetin, resveratrol, EGCG, curcumin, carotenoids, organic sulfur compounds, phytoestrogens, etc.), PFAs, probiotics, prebiotics, FMHS, vitamins, and minerals. It is recommended to choose olive oil and tea oil with high levels of PFAs for cooking or as added oils for food, and take probiotic products. Furthermore, diets that have a regulatory effect on hypertension, such as the DASH, MD, and KD, also follow the principle of including foods rich in the aforementioned components. The mechanism by which these foods or dietary patterns regulate blood pressure manifests as increasing nitric oxide activity, inhibiting the release of inflammatory factors, regulating sodium–potassium balance, promoting the growth of intestinal probiotics, and maintaining the stability of the intestinal barrier, all of which aim to achieve the goal of regulating blood pressure.

Finally, it is worth noting that the intestinal barrier function affected by a high-salt diet has the ability to recover. The related dietary therapies, such as supplementing probiotics, prebiotics, and adjusting the diet structure, were all related to the restoration of the intestinal barrier. Paying attention to the intestinal barrier has significant reference value for the treatment of hypertension and other diseases. Firstly, from a clinical perspective, by integrating biotechnology and drug efficacy, targeted development of drugs for repairing the intestinal barrier of hypertensive patients should be carried out, enabling the drugs to directly act on the intestinal barrier. This approach can lower blood pressure while reducing the side effects of current antihypertensive drugs. Secondly, supplementing FMHS is an important part of hypertension dietary therapy. However, the rational combination and efficacy of these substances still need to be explored. Nevertheless, the advantages of multi-component and multi-target synergistic effects compared to single components deserve the attention of researchers and application in disease control. In the future, based on the concept of precision nutrition, for hypertensive patients, health foods or foods with antihypertensive effects derived from FMHS can be developed. The antihypertensive efficacy of the developed products should be scientifically evaluated. Through component research and efficacy evaluation based on intestinal barrier repair, the antihypertensive products with FMHS can be made to have both efficacy and safety, enriching non-pharmacological treatment methods for hypertension.

## Figures and Tables

**Table 1:** Mean urinary sodium levels of Chinese residents by age group (2019).

Age	Sex	Mean Sodium Excretion (mmol/24 h)	Mean Salt Intake (g/24 h)
3~6	male/female	86.99	5.09
6~16	male/female	151.09	8.84
>16	male	194.76	11.39
	female	181.54	10.62

**Table 2:** The impact of a high-salt diet on the abundance of gut microbiota and the host.

Microorganism (Genus/Species)	Variation Tendency	Observed or Proposed Host Effect	References
*Prevotella*	Increased	Increased *Prevotella* abundance was positively correlated with intestinal mucosal inflammation and could stimulate Th17 cell-mediated immune responses.	[46,47]
*Weissella*	Decreased	Reduced *Weissella* abundance might impair lactic acid production, which typically inhibits foodborne pathogens.	[46,48]
*Clostridium* (e.g., *C. perfringens*, *C. difficile*)	Increased; *Clostridium* species converted primary bile acids into secondary bile acids.	Increased abundance of *Clostridium* species can cause severe infections and has been linked to liver cancer progression through the conversion of primary to secondary bile acids, which might inhibit immune response.	[49,50,51]
*Catenibacterium*	Increased	Higher *Catenibacterium* abundance was observed in polycystic ovary syndrome patients and was associated with a more diverse gut microbiota, with variations among ethnic minority populations (e.g., Li ethnic group) in China.	[49,52,53]
*Klebsiella*	Increased	Increased *Klebsiella* was prevalent in hypertensive patients; its cell wall LPS could regulate the immune system and contribute to intestinal inflammation.	[49,54,55]
*Mogibacteriaceae*	Increased	Changes in the abundance of intestinal mucosa *Mogibacteriaceae* in rectal cancer patients might be related to disease onset.	[49,56]
*Novosphingobium*	Increased; Its dihydrolipoamide acetyltransferase component (PDC-E2) protein showed high homology to human PDC-E2.	The subordinate strain’s PDC-E2 protein had high homology with the immunodominant region of human PDC-E2, suggesting a possible role in primary biliary cirrhosis.	[49,57]
*Chryseobacterium*	Increased	Most strains were drug-resistant; could cause severe infections (e.g., bacteremia, pneumonia, meningitis) in immunocompromised individuals. Indole-producing strains, while less virulent, also contributed to these diseases.	[49,58,59]
*Lactobacillus*	Decreased	Decreased *Lactobacillus* abundance may impair intestinal flora balance, reduce protease secretion (neutralizing bacterial toxins), and compromise intestinal barrier function.	[60]
*Clostridium XIVa*	Decreased	Reduced *Clostridium XIVa* abundance, a probiotic with properties aiding intestinal microecological balance, was less prevalent in ulcerative colitis patients.	[60,61]
*Pseudoflavonifractor*	Decreased	Reduced *Pseudoflavonifractor* abundance, a core gut microbiota, has been considered a biomarker for obesity in recent years.	[60,62]
*Alistipes*	Increased	Subordinate strains were isolated from patients with appendicitis, abdominal and rectal abscesses, and rectal cancer, indicating a key role in inflammation.	[60,63,64]
*Parasutterella*	Increased	Changes in *Parasutterella* abundance were linked to metabolic disorders; it helped maintain bile acid homeostasis and regulated cholesterol metabolism.	[60]
*Akkermansia*	Increased	*Akkermansia*, a probiotic, could degrade mucoprotein substrates produced by the host.	[60]
*Ruminococcus* (e.g., *R. gnavus*)	Increased; *R. gnavus* produced a substance that causes DC cells to produce inflammatory cytokines.	This bacterial community, including *R. gnavus*, was capable of degrading resistant starch and cellulose. *R. gnavus* produced inflammatory cytokines (e.g., TNF-α), linking the intestinal bacterial community to extraintestinal inflammatory diseases.	[41,65]
*Oscillospira*	Increased; The strain was less abundant in patients with inflammatory bowel disease.	Increased *Oscillospira* abundance might aid in the formation of secondary bile acids and resistance to *Clostridium difficile* infections, despite being less abundant in patients with inflammatory bowel disease.	[41,66,67]
*Roseburia* (e.g., *R. intestinalis*)	Increased	*Roseburia*, including probiotic strains like *R. intestinalis*, produced butyric acid in the colon.	[41,67,68]
*Anaerostipes*	Decreased	Reduced *Anaerostipes* abundance, a probiotic that converted lactic acid, acetic acid, and sugar into butyric acid in the intestines.	[69]

**Table 3:** Anti-hypertensive FMHS and their mechanisms.

Substances	Active Ingredients	Antihypertensive Mechanism	References
*Mori folium*	/	Increased NO activity.	[130]
*Semen raphani*	/	Regulation of NOS expression.	[131]
*Crataegi fructus*	Hawthorn flavonoids	Inhibited oxidative stress in blood vessels.	[132]
*Puerariae lobatae radix*	Flavonoids, puerarin	Expanded blood vessels and improved microcirculation.	[133]
*Panax ginseng*	Ginsenoside	Regulation of NOS expression.	[133]
*Semen cassiae*	/	Promoted eNOS expression, antioxidant activity, and inhibition of angiotensin-converting enzyme (ACE) activity.	[134]
*Dendranthema morifolium*	Luteolin, etc.	Inhibit AngII and NF-κB pathways.	[135]
*Lycium chinese Miller*	Quercetin, betaine, etc.	Interfered with multiple signaling pathways (AKT1, EGFR, MYC, etc.).	[136]
*Fructus mume*	/	Acted on vascular smooth muscle cells(VSMC) to protect blood vessels.	[137]
*Polygonati odorati*	Flavonoids	Inhibited vascular oxidative stress.	[138]
*Cannabisfructus*	*Hemp seed oil*	/	[139]
*Ginkgo biloba* L.	Bilobalide	Increased the activity of superoxide dismutase in the serum and reduced the concentration of malondialdehyde in the serum.	[140]
*Eucommia ulmoides* Oliv.	Chlorogenic acid	Improved endothelial cell function. Inhibited oxidative stress; regulated mitochondrial dysfunction, etc.	[141]

## Data Availability

Not applicable.
